# Convergent Evaluation of Working Memory and Arithmetic Ability in a Child with Autism Spectrum Disorder without Intellectual Impairment

**DOI:** 10.3389/fpsyg.2017.01278

**Published:** 2017-07-26

**Authors:** Sandra Pellizzoni, Maria C. Passolunghi

**Affiliations:** Department of Life Science, University of Trieste Trieste, Italy

**Keywords:** autism, arithmetics, math ability, atypical development, working memory

## Abstract

Studies focusing on a joint evaluation of both Working Memory (WM) and Math Ability (MA) in autism are far from abundant in literature, possibly due to inadequate methodological approaches and reported inconsistencies between results obtained in each separate field of research, resulting in contradictory conclusions. The specific aim of this case report is therefore evaluating and integrating results on these two cognitive abilities in a child with autism spectrum disorder without intellectual impairment. Our data on an autistic 10-year-old child (M.N.) show that the levels of functional (active vs. passive), rather than structural (phonological vs. visual), data manipulation are quite relevant in the way the child scored differently in the various tasks. Furthermore, M.N. generally displayed average to good ability levels in math calculation, except for oral multiplication, and division activities. By way of conclusion, data are discussed in terms of strengths and weaknesses in relation to special learning trajectories in education and the relevant achievements.

## Introduction

According to Baddeley's model (Baddeley and Hitch, 1974; Baddeley, 1986) and subsequent adaptations (Gathercole and Alloway, 2006), memory may be divided into two components, namely a more passive one, also known as short-term memory (STM), which is dedicated to the retention of transient information stored in the phonological loop and visuo-spatial sketchpad; and a more active one, also known as working memory (WM), which corresponds to the central executive and is involved in information elaboration. Memory plays a relevant role in the development of math-related skills (Alloway and Passolunghi, 2011; Friso-van den Bos et al., 2013; Peng et al., 2016), and, more specifically, in the following two main aspects: (1) elaboration of phonological and visuo-spatial components, and (2) level of information manipulation (passive vs. active processes). Although the distinction between phonological and visuo-spatial components of memory has been largely investigated in the literature with regard to typical development (Baddeley, 1986, 2000, 2002), the volume of data on the active vs. passive distinction is still limited (Cornoldi and Vecchi, 2003; Gathercole and Alloway, 2006; Passolunghi and Cornoldi, 2008), especially in relation to autism. It is our belief that a WM evaluation for each component is crucial, as it allows researchers to separately investigate the phonological and visuo-spatial component, on the one hand, and the active manipulation and transformation of data while recalling them, on the other. The latter model, evaluating passive and active component of WM, is observed through tasks that require recalling data in the same format in which they are presented (i.e., passive tasks: the subject is asked to recall a list of numbers, patterns, or positions in the same order they were presented), or through tasks that require multi-level management of incoming data (e.g., The Listening Span Test, Daneman and Carpenter, 1980).

Effects on memory at componential and manipulation level were tested through an analysis tool specifically developed to investigate the arithmetic skills displayed by a child with autism spectrum disorder without intellectual impairment. Our goal was to yield results able to provide a fine-grained evaluation of the relevant component- and process-related aspects underlying WM performance. We found that children with autism spectrum disorder without intellectual impairment are far from numerous among our patients, and that high-level cognitive tests are quite demanding for autistic children with average intellectual abilities.

## Background

Research on WM and math abilities (MA) in children with autism still accounts for a limited share of studies in the literature, although investigations carried out on typical development clearly show a significant correlation between these two variables (Alloway and Passolunghi, 2011; Friso-van den Bos et al., 2013; Peng et al., 2016). This knowledge gap may be due to (1) reported inconsistencies in studies on WM in autism (Kercood et al., 2014), (2) divergent results in studies on MA in autism (Meyer and Minshew, 2002; Mayes and Calhoun, 2006; Iaculano et al., 2014), and (3) an overall paucity of scientific work on componential aspects of MA in relation to this pathological condition (Titeca et al., 2015).

A recent review of the literature on memory in autism shows that research published so far has focused mainly on componential aspects: children with autism seem to be as likely as typical children to activate articulatory rehearsal, despite performing poorly in spatial tasks (Kercood et al., 2014). However, the active/passive paradigm in autism remains largely understudied. Our case report aims at affording new insight to the scientific community that may help fill this knowledge gap, by providing a comprehensive picture of calculation abilities in an individual with autism spectrum disorder without intellectual impairment (hereafter M.N.) through both a set of math-related tasks, specifically aimed at testing arithmetic skills, and a set of WM tasks covering different processes (passive vs. active) and components (phonological vs. visuo-spatial). Our testing method aims at yielding results that may indicate a relation—if any—between the type of material or process and the child's performance. On the basis of the results obtained by Kercood et al. (2014), our hypothesis was that M.N. would perform better in tasks involving the verbal component and worse in tasks involving the visuo-spatial component. Moreover, as far as the passive vs active process debate is concerned (Gathercole and Alloway, 2006), we expected M.N. to perform very well in WM tasks requiring a passive manipulation of data (e.g., Digit Span Forward test), and to perform averagely well in tasks requiring an active manipulation of data (e.g., Listening Span test), independently of the componential level of analysis. This hypothesis is based on previous studies carried out on children with typical as well as atypical development and their manipulation of memorized pieces of information (Passolunghi and Cornoldi, 2008).

As regards arithmetic skills, we expected M.N. to solve simple oral arithmetical calculations, like subtractions or additions, quite effortlessly, as such tasks largely depend on a passive component of memory, i.e., effective memory storage or Short Term Memory (Gathercole and Alloway, 2006). Conversely, M.N. was expected to score sensibly lower in active manipulation of data, such as the one involved in oral division and multiplication operations, given the cognitive challenge of successfully performing complex semantic representations of data (Gathercole and Alloway, 2006). Finally, written operations, which allow for a lower level of memory load regardless of the complexity of multi-digit figures, were expected to yield average test scores.

At the time of the study M.N. was a fourth-grader of 10 years and 9 month of age. He was diagnosed with autism at 3 years of age, on the basis of criteria illustrated in the DSM IV-TR (American Psychiatric Association, 2000), resulting from two structured research diagnostic instruments administered by a team of specialized professionals including a neuropsychologist, a psychologist, and an educator. Autism Diagnostic Interview Revised tool (Lord et al., 1994) showed a qualitative impairments in (1) Reciprocal social interaction (score 20, cut-off-10), (2) Communication (verbal score 16, cut-off-8, non-verbal score 9 cut-off-7), (3) Repetitive behaviors and stereotyped patterns (score 4, cut-off-3), the child showed the abnormality development before 36 months. The Autism Diagnostic Observation Schedule (ADOS) showed an algorithm score 20 (Lord et al., 2000). General intelligence was tested tool trough the WISC-IV tool (Wechsler, 2003), which resulted in a synthetic score of 89^1^. M.N.'s profile displays significant discrepancies, particularly between verbal comprehension (lowest score) and perceptual reasoning (higher score). Furthermore, M.N. obtained average scores in relation to Working Memory and Processing Speed Indexes. Indeed, he performed averagely in all tasks, except for Block Design and Picture Completion (above average), Vocabulary and Matrix Reasoning (below average), and Comprehension (extremely below average).

We put together a control sample comprising twenty 10-year-old children (mean age: 122.25 months; 11 boys and 9 girls) with Typical Development (T.D.). Evaluation was carried out at the end of fourth grade. Parents (both mothers and fathers) were asked to sign an informed consent to (1) authorize their children's participation in the study and (2) authorize the publication of the research report.

### Measures

#### Memory tasks

The following tests were carried out: Digit Span Forwards task (Wechsler, 2003); Listening Span task (Daneman and Carpenter, 1980); Visual Pattern task (Della Sala et al., 1997); and Counting Span task (Siegel and Ryner, 1989).

#### Calculation tasks

1. AC-MT (Cornoldi et al., 2002). The AC-MT is a standardized mathematics test designed for pupils in fourth grade. This test is well-known in the Italian context and used in both clinical and educational settings. It allows for the evaluation of calculation and number comprehension skills through collective as well as individual testing. AC-MT test indexes are divided as follows: (a) Written Calculation, (b) Numerical Knowledge, (c) Accuracy, and (d) Speed. The test concurrent validity, reported in the AC-MT manual, is 0.84, and is therefore deemed satisfactory (Cornoldi et al., 2002). The scores concerning reliability are expressed as follows: (a) Written Calculation: 0.60, (b) Numerical Knowledge: 0.79, (c) and (d) Accuracy and Speed: 0.85.
2. Evaluation of Math Abilities (Giovanardi Rossi and Malaguti, 1998). Specific tasks from this tool were selected, namely written and oral calculation tasks (additions, subtractions, multiplications, and divisions). This test tool allowed for an integration of the AC-MT tool, which lacks evaluation of oral multiplication and division.

### Procedure

Both the case and the control group were tested with the same tools, except for tests, for which a standardization for the Italian population already exists. Test administration was scheduled in a series of three sessions: session 1—AC-MT (Cornoldi et al., 2002); session 2—Evaluation of Math Abilities (Giovanardi Rossi and Malaguti, 1998) and Digit Span Forward test (WISC-IV, Wechsler, 2003); session 3—Listening Span Test (Daneman and Carpenter, 1980); Visual Pattern Test (Della Sala et al., 1997); and Counting Span Test (Siegel and Ryner, 1989).

## Results

M.N.'s memory abilities were evaluated through tasks testing WM-related componential and manipulation levels using Digit Span Forward, Listening Span, Visual Pattern Test, and Counting Span Test. M.N.'s performance can be described as follows: compared to the typical development (T.D.) group, M.N. was better at remembering numbers in the Digit Span Forward test [*t*_(19)_ = 3.67, *p* = 0.002], scoring a 99.92 percentile (95% CI: 99.36–100.00). In the Listening Span test M.N.'s performance was within the average [*t*_(19)_ = 0.15, *p* = 0.880]. M.N.'s score was approximately 55.98 percentile (95% CI: 38.71–72.42), with respect to the T.D. distribution.

The visuo-spatial sketchpad was carried out via the Visual Pattern test, whose indexes yielded significant results: M.N. scored a 99.87 percentile (95%: CI 99.04–100%) in the matrix test [*t*_(19)_ = 3.46, *p* = 0.003], and a 99.77 percentile (95% CI: 98.44–100%) in the box test [*t*_(19)_ = 3.21, *p* = 0.005], with respect to the T.D. group. M.N. obtained an average score with respect to the control group in the Counting Span Task [*t*_(19)_ = 0.39, *p* = 0.18] as shown in Table 1.

**Table 1:** Scores obtained by M.N. and the T.D. group in WM tasks.

**Componential level**	**Manipulation level**		**Score**	
**Tasks**	**Active**	**Passive**	**M.N. Score**	**T.D. Mean Score (*****SD*****)**
**WM- PHONOLOGICAL LOOP**				
Digit span forward (Wechsler, 2003)		x	12^**^	8.2 (1.01)
Listening span (Daneman and Carpenter, 1980) (words correctly remembered)	x		21	18.60 (2.5)
**WM VISUO-SPATIAL SKETCHPAD**				
Visual pattern task—matrix (Della Sala et al., 1997)		x	28^**^	20.05 (2.24)
Visual pattern task—box (Della Sala et al., 1997)		x	182^**^	148.8 (10.10)
Counting span task (Siegel and Ryner, 1989)	x		5	3.75 (0.80)

^*^p < 0.05;

** p < 0.01;

^***^p < 0.001.

The MA profile of M.N. was outlined using the AC-MT tool (Cornoldi et al., 2002) and Evaluation of Math Abilities tool for fourth grade (Giovanardi Rossi and Malaguti, 1998). M.N.'s performance was subsequently compared with the Italian population norms. The scores obtained by M.N. are in line with his grade's average. More specifically, M.N. reached the 50 percentile in “Written calculation in class”, 50 percentile in “Numerical knowledge”, 50 percentile in “Accuracy”, and between 50 and 60 percentile in terms of “Total time” as shown in Table 2.

**Table 2:** Scores obtained in terms of AC-MT (Cornoldi et al., 2002) parameters for MA.

	**M (*SD*)**	**M.N.'s score**
Written calculation (correct answers)	6.62 (1.47)	7
Numerical Knowledge (correct answers)	18.09 (3.63)	18
Accuracy (numbers of errors)	5.69 (4.43)	5
Total Time (seconds)	130.77 (53.52)	118

M.N.'s arithmetic written and oral skills were assessed using the Evaluation of Math Abilities tool (Giovanardi Rossi and Malaguti, 1998): M.N. performed well in addition and subtraction in oral calculation (total score 13/13), and insufficient in oral multiplication and division (total score 6/10). In Written calculation M.N. performed well in addition and subtraction (total score 8/8) and discretely in multiplication and division (total score 6/8). All results are shown in Table 3.

**Table 3:** Scores obtained by M.N. in “Evaluation of Math Abilities” test (Giovanardi Rossi and Malaguti, 1998).

	**Oral calculation**		**Written calculation**	
	**Score**	**Mark**	**Score**	**Mark**
Addition	13/13	Good	8/8	Good
Subtraction	13/13	Good	8/8	Good
Multiplication	6/10	Insufficient	6/8	Discrete
Division	6/10	Insufficient	6/8	Discrete

## Discussion

WM and MA have been widely investigated in typical development, but only sporadically in subjects with autism. The present study is an attempt at shedding some light on this topic, by providing an account of both mathematical-ability- (Titeca et al., 2015) and working- memory-related (Gathercole and Alloway, 2006) components, and the processes that lie at the basis of the connection between the two. In this study we analyzed the calculation abilities of a child with autism spectrum disorder without intellectual impairment (M.N.) and an average intelligence level, with the goal of testing his arithmetic abilities in connection with WM performance.

### Memory: componential and manipulation level analysis

In WM-related tasks, M.N. showed a high-level performance when tasks required a passive management of data (e.g., Digit Span Forward and Visual Pattern Tests), independently of WM components involved. On the other hand, when tested on complex manipulation tasks (e.g., Listening Span and Counting Span), M.N. scores do not differ from those achieved by T.D. children, independently from the type of data to be processed. Results thus obtained suggest that the level of data manipulation implicated in the tasks resolution, rather than the type of component, seems to determine the degree of difficulty for M.N. This result seems to corroborate data underlining the importance of elaboration level when evaluating WM in autism, while partially invalidating claims concerning the role of the type (visual vs. phonological) of material proposed (Kercood et al., 2014). Indeed, when asked to report strategies used on passive level manipulation tasks (Digit Span Forward), M.N. stated the following: “…I listen to the numbers you utter, then I close my eyes and I see them in my mind. They are in a row […]. They stand there, waiting for me to read them to you and then vanish, and when the new ones arrive, they form another line. It's easy because I can see them.” In terms of strategies, therefore, M.N. seemed to prefer visual imagery, thus confirming a well-known theory in the literature (Mitchell and Ropar, 2004).

### Manipulation level and arithmetical operations

M.N. confirmed our hypothesis as he obtained good results in subtraction and addition oral calculation (Giovanardi Rossi and Malaguti, 1998). Our hypothesis was further corroborated by his “total time” score, which refers specifically to oral calculation speed, as well as the mean score he obtained in the accuracy parameter. This seems to indicate that M.N.'s ability may depend mainly on effective memory storage, which requires a passive level of manipulation of incoming information.

Accordingly, M.N.'s poor performance in oral divisions and multiplications may indicate that active manipulation of data represents a significant factor when evaluating the task complexity in subjects with autism. Even with a medium level of WM performance, these two types of tasks appear more demanding in their oral form compared to the written one. However, M.N. only made show four errors in the 10 tasks, that which may indicate that he is not completely unable to perform the above-mentioned calculations, but simply that the oral performance is the real difficulty, as he was able to perform the self-same calculations when written, and therefore requiring lower memory storage.

Literature on typical development suggests that multiplications and divisions require more complex and cognitively challenging operations. Having no direct access to any memorized solution or use of linear number representation, M.N. had to solve multiplications and divisions by activating his procedural knowledge, which requires the use of semantic representations that may result in an increased cognitive effort and, consequently, potential WM overload.

When considering written operations, on the other hand, M.N. performed quite well in written addition and subtraction operations, and discretely in multiplication and division operations, as evaluated trough Giovanardi Rossi and Malaguti (1998). Writing down each single operation may have reduced M.N.'s WM load, especially with regard to division and multiplication operations, thus allowing for a correct solution of the tasks.

In the qualitative questionnaire administered at the end of the task, M.N. himself provides corroborating evidence of the greater amount of cognitive load experienced in solving these tasks: indeed, when commenting the testing process, he stated that “…it's easy because when you have to add or subtract, numbers appear in line. It's easy to go back and forth on one line to add or subtract […]. When multiplying I have the big tables but…they are not always useful as they don't work every single time. […] How do you do the 18 table? I need pen and paper and I write it down…I cannot remember it quickly. Unless I take the 9 table and double it, it's difficult […]. When I am sloppy and write down the numbers in the wrong column, I get it all wrong.” The appropriate use of visual strategies helps M.N. solve only specific kinds of arithmetic tasks, such as subtraction and addition, in a quick, and accurate manner. These strategies seem to lie at the basis of the ability to correctly solve calculation tasks, especially when they are linear in nature. The self-same strategies, however, do not compensate for M.N.'s impaired ability of tackling problems that may result in an excessive load of the WM function (such as oral divisions or multiplications).

In conclusion, M.N.'s MA seems to be closely related to his WM skills. We are aware of the fact that functioning of children with autism spectrum disorder without intellectual impairment may largely depend on the age at which the ability is assessed (Titeca et al., 2015), as well as on and other cognitive functions such as intelligence level (Mayes and Calhoun, 2003) of executive function nature (De Smedt et al., 2013); moreover, we are aware that a case study cannot be universally applied to children in special educational settings or children with different intelligence levels. We do, however, believe that our study may contribute to enhancing our knowledge of the relation between calculation and memory abilities, while providing some insight into children's strategies that are conducive of an implementation of memory skills, and, as a result, of calculation abilities too, both in educational settings and in everyday life.

## Author contributions

SP designed the study, drafted a first version of work and provided a final approval of the manuscript. MP supervised a critical revision of the work and proposed the final approval of the version to be published.

### Conflict of interest statement

The authors declare that the research was conducted in the absence of any commercial or financial relationships that could be construed as a potential conflict of interest.

## Footnotes

^1^M.N.'s score breakdown: VCI = 85; PRI = 103; WMI = 89; PSI = 92.
